# Gut microbiota profile in newly diagnosed pulmonary tuberculosis patients: an exploratory pilot study in southern India

**DOI:** 10.1186/s13099-025-00736-x

**Published:** 2025-08-11

**Authors:** Tejaswini Baral, Shaik Mohammad Abdul Fayaz, Mohan K. Manu, Chandrashekar Udyavara Kudru, Jitendra Singh, Chiranjay Mukhopadhyay, Mahadev Rao, Kavitha Saravu, Sonal Sekhar Miraj

**Affiliations:** 1 Department of Pharmacy Practice, Manipal College of Pharmaceutical Sciences, Manipal Academy of Higher Education, Manipal, 576104 Karnataka India; 2 Department of Biotechnology, Manipal Institute of Technology, Manipal, Manipal Academy of Higher Education, Manipal, 576104 Karnataka India; 3 Department of Respiratory Medicine, Kasturba Medical College, Manipal, Manipal Academy of Higher Education, Manipal, Karnataka 576104 India; 4 Department of General Medicine, Kasturba Medical College, Manipal, Manipal Academy of Higher Education, Manipal, Karnataka 576104 India; 5 Department of Translational Medicine, All India Institute of Medical Sciences, Bhopal, Madhya Pradesh 462020 India; 6 Department of Microbiology, Kasturba Medical College, Manipal, Manipal Academy of Higher Education, Manipal, Karnataka 576104 India; 7 Department of Infectious Diseases, Kasturba Medical College, Manipal, Manipal Academy of Higher Education, Manipal, Karnataka 576104 India; 8 Centre for Public Health Pharmacy, Department of Pharmacy Practice, Manipal College of Pharmaceutical Sciences, Manipal Academy of Higher Education, Manipal, Karnataka 576104 India

**Keywords:** Antitubercular therapy, Gut microbiota, Probiotics, Pulmonary tuberculosis, 16S amplicon sequencing

## Abstract

Emerging evidence suggests the link between pulmonary tuberculosis (PTB) and gut microbiota dysbiosis. This is the first study from the southern Indian population that characterized the gut microbiota of PTB patients using 16 S amplicon sequencing. The analysis revealed a significant reduction in gut microbial diversity among PTB patients, with particularly lower alpha diversity (Chao1 index, *p* ≤ 0.0001) than healthy controls (HC). This was further depleted during antitubercular therapy (ATT). Beta diversity indicated distinct clustering in all the groups (*p* < 0.05). Subgroup analyses showed that supplementation of probiotics with ATT improved microbial richness and diversity. However, broader shifts in composition were not observed. At the genus level, specific taxa were upregulated or downregulated in PTB patients compared to HC. Functional analysis showed a depletion in biosynthesis pathways in PTB patients. Short-term probiotic supplementation had a partial effect on microbial recovery but did not fully restore gut microbial diversity. These findings highlight persistent dysbiosis in PTB patients, even after ATT. Large-scale studies are needed to evaluate the role of microbiome-targeted therapies to address this dysbiosis.

**Supplementary Information:**

The online version contains supplementary material available at 10.1186/s13099-025-00736-x.

## Introduction

Tuberculosis (TB) is among the most widespread infectious illnesses, inflicting considerable distress globally. *Mycobacterium tuberculosis* (*Mtb*) is the sole etiological agent of TB, primarily targeting the lungs, referred to as pulmonary tuberculosis (PTB). However, it may also impact other organs. As per the Global TB Report 2024, the cases of TB were 10.8 million in 2023 [1]. In 2023, a global total of 8.2 million individuals were newly diagnosed with TB, an increase from 7.5 million in 2022 [1]. Five nations represented 56% of the worldwide TB cases [1]. Among these, India contributes 26%, followed by Indonesia (10%), China (6.8%), the Philippines (6.8%), and Pakistan (6.3%). In 2023, 1.09 million fatalities worldwide were formally attributed to TB and recorded. Among HIV-negative persons, India alone accounted for 29% of the worldwide total of TB-caused deaths [1].

The gut microbiota comprises many microbes, including prokaryotes, archaea, viruses, and microbial eukaryotes [2]. The gut and lung axis works through bidirectional microbial communication between the gut and lungs. The link between PTB and gut microbiota dysbiosis is an emerging research area. The pleiotropic effect of gut microbiota has illuminated the possibility of its influence on TB [3]. Unlike genetic epidemiology, the microbiome is far more amenable to modification than the human genome, as several host and environmental factors can influence and reshape it. Thereby enabling its alteration to be harnessed for a wide range of therapeutic applications [4]. Advancements in next-generation sequencing technologies have enhanced our understanding of the gut microbiota in TB [5, 6].

Previous studies have reported that probiotics may help alleviate anti-tuberculosis treatment (ATT)-induced adverse drug reactions, particularly during the intensive phase of therapy, making them of clinical interest in TB management [7, 8]. In a subset of enrolled patients in our study, probiotic supplements were prescribed for good gut health during (ATT) based on the treating physician’s’ discretion and hospital formulary availability. This allowed for an exploratory evaluation of how probiotics supplementation might influence gut microbiota in PTB patients undergoing ATT. In this study, we aimed to characterize the gut microbiota of PTB patients from the southern Indian population in terms of microbial diversity, marker taxa identification, and inferred microbial functionality.

## Methodology

### Study setting and population

This pilot observational study was conducted in a tertiary care hospital in Manipal, Coastal Karnataka, India, from January 2022 to December 2024. In the study, a total of 32 participants were enrolled (Supplementary File 1; Figure S1). Among them, 20 were microbiologically confirmed newly diagnosed PTB patients. Participants who were pregnant, aged < 18 or > 80 years, had gastrointestinal diseases, liver disease, retroviral diseases, autoimmune diseases, intake of antibiotics or probiotics during the last two months, and those who were on immunosuppressants were excluded. By matching age and gender, 12 healthy controls (HCs) were enrolled, which served as a reference standard for normal gut microbial communities. HCs are non-TB individuals. The demographics of the participants selected are given in Supplementary File 1. From 20 selected PTB patients grouped as PTB_before_ATT, whose fecal samples were collected before ATT initiation. Additionally, nine follow-up fecal samples were collected from the participants within two months of initiating anti-tuberculosis treatment (ATT). These samples were considered for subgroup analysis (Supplementary File 1; Figure S1). Five participants received probiotic supplementation (VSL3, *n* = 2; Evogut, *n* = 3) for good gut health, grouped as PTB_after_ATT_Probiotics, and the remaining four samples were grouped as PTB_after_ATT. The samples were processed at the Bencos Research Solutions Pvt. Ltd, Maharashtra, India, and stored at -80 °C until sequencing and analysis.

### DNA extraction and 16 S sequencing

Extraction of genomic DNA was performed using QIAmp PowerFecal Pro DNA Kit (Qiagen) using the manufacturer’s protocol [9]. For library preparation, the V3-V4 hypervariable regions of the 16 S gene were amplified using primers (341 F: CCTACGGGNGGCWGCAG and 805R: GACTACHVGGGTATCTAATCC), including overhang adapter sequences for Illumina sequencing, as per the manufacturer’s instructions (Illumina, San Diego, CA, USA). Genomic DNA with primers was lodged onto the 96-well plate, and PCR was performed in a thermal cycler to obtain the amplicon PCR. According to the protocol followed by Illumina for sequencing, the quality of the PCR product was analyzed using a Bioanalyzer DNA 1000 chip, which gave a ~ 550 bp trace of amplicon on the Bioanalyzer (Agilent Technologies 2100 Bioanalyzer). In preparation for cluster generation and sequencing, indexed and purified pooled libraries were denatured with NaOH, diluted with hybridization buffer, and then heat denatured before sequencing on Illumina NovaSeq6000. Raw sequencing reads were deposited in the National Center for Biotechnology Information Sequence Read Archive (BioProject ID PRJNA1207258).

### Bioinformatics analysis

Preprocessing analysis was performed to trim the primers’ sequences 341 F and 805R, which were utilized for the amplicon sequencing. Primers were removed using cutadapt (v4.9) [10]. The quality of the reads was verified, and the reads were eliminated using the fastp (v0.23.2) [11].

The Divisive Amplicon Denoising Algorithm 2 (DADA2) (v1.32.0) package in R (v4.4.1) was used to perform the Amplicon Sequence Variants (ASVs) analysis [12]. The DADA2 pipeline builds an error model from the input data to infer the true biological sequences from the observed data, a process called denoising. During this process, sequences related to mitochondria and chloroplasts were also removed to focus on the target sequences. The denoising statistics, including the number of input reads and the percentage of surviving reads after filtration and chimera removal, are provided in Supplementary file 2.

The resulting ASVs were aligned to the SILVA database (v138) for the taxonomy assignment. The DADA2 package utilizes a native implementation of the naive Bayesian classifier method for this purpose. The assign taxonomy function takes as input a set of sequences to be classified and a training set of reference sequences with known taxonomy, and outputs taxonomic assignments with at least minBoot bootstrap confidence [13].

Sequences were rarefied before calculating alpha and beta diversity statistics. Rarefaction was applied at a depth of 1103 reads, and samples with fewer than 1103 sequences were excluded from diversity analyses (Supplementary File 1 (Figure S2 and Figure S3) shows the rarefaction curves). As a result, two samples from the PTB_before_ATT group (SC021-01 and SC030-01) were excluded from the overall group alpha and beta diversity analyses, reducing the group size from *n* = 20 to *n* = 18. Rarefaction was not performed to retain all paired samples in the subgroup comparison between PTB_before_ATT and PTB_after_ATT_Probiotics (*n* = 5) for the diversity analysis. Since these two baseline samples (SC021-01 and SC030-01) corresponded to patients prescribed probiotic supplementation, exhibited low sequencing depth and would have been excluded by rarefaction.

The sequences were estimated using a common non-parametric statistical Kruskal-Wallis test. The observed taxa, Chao1, and Shannon index were estimated for alpha diversity. Bray-Curtis and weighted Unique Fraction Metric (UniFrac) metrics were used to analyze beta diversity. Principal Coordinate Analysis (PCoA) was used to visualize the results. The significance of the microbial community differences among groups was evaluated using the Permutational Multivariate Analysis of Variance (PERMANOVA).

Differential abundance analysis was performed for Linear Discriminant Analysis (LDA) Effect Size (LEfSe). R package microbiomeMarker (v1.8.0) [14] was used for LDA effect size (LEfSe) analysis to identify differentially abundant taxa. Non-parametric factorial Kruskal-Wallis sum-rank test to detect features with significant differential abundance with respect to the class of interest.

Phylogenetic Investigation of Communities by Reconstruction of Unobserved States (PICRUSt2) (v2-2.5.3) [15] was used to predict the microbial functional profiles based on the marker gene sequences, and ANOVA-like differential expression **(**ALDEx2) (v1.36.0) [16] was used to perform compositional data analysis and check the estimated effect size range.

### Ethical approvals

This study was approved by the Kasturba Medical College and Kasturba Hospital Institutional Ethics Committee [IEC: 761/2021], and informed consent was obtained from all participants. The study was registered in the Clinical Trial Registry of India (CTRI/2022/01/039335).

## Results

### Taxonomical composition of gut microbiota

**Fig. 1:**
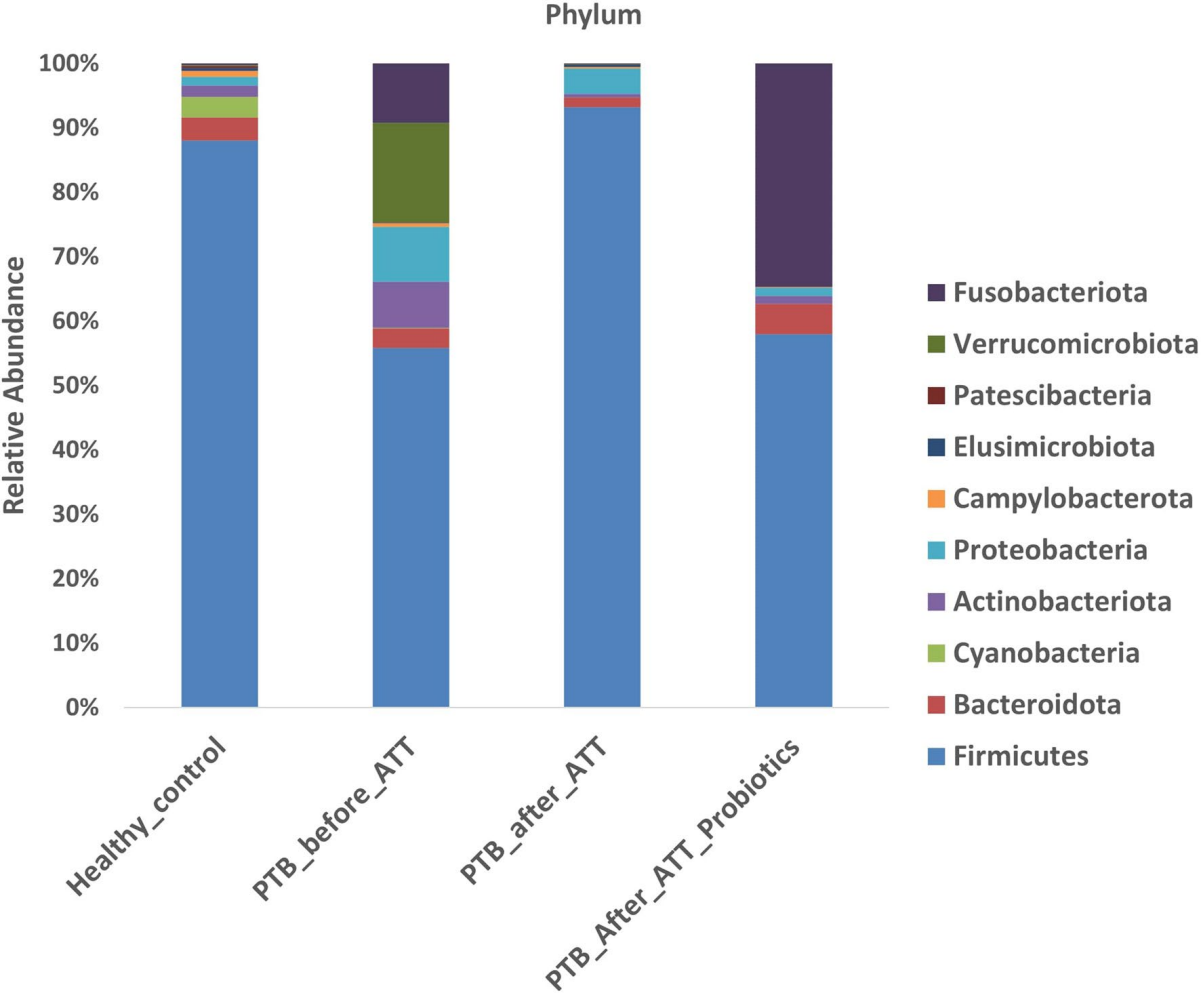
Relative abundance at phylum level

**Fig. 2:**
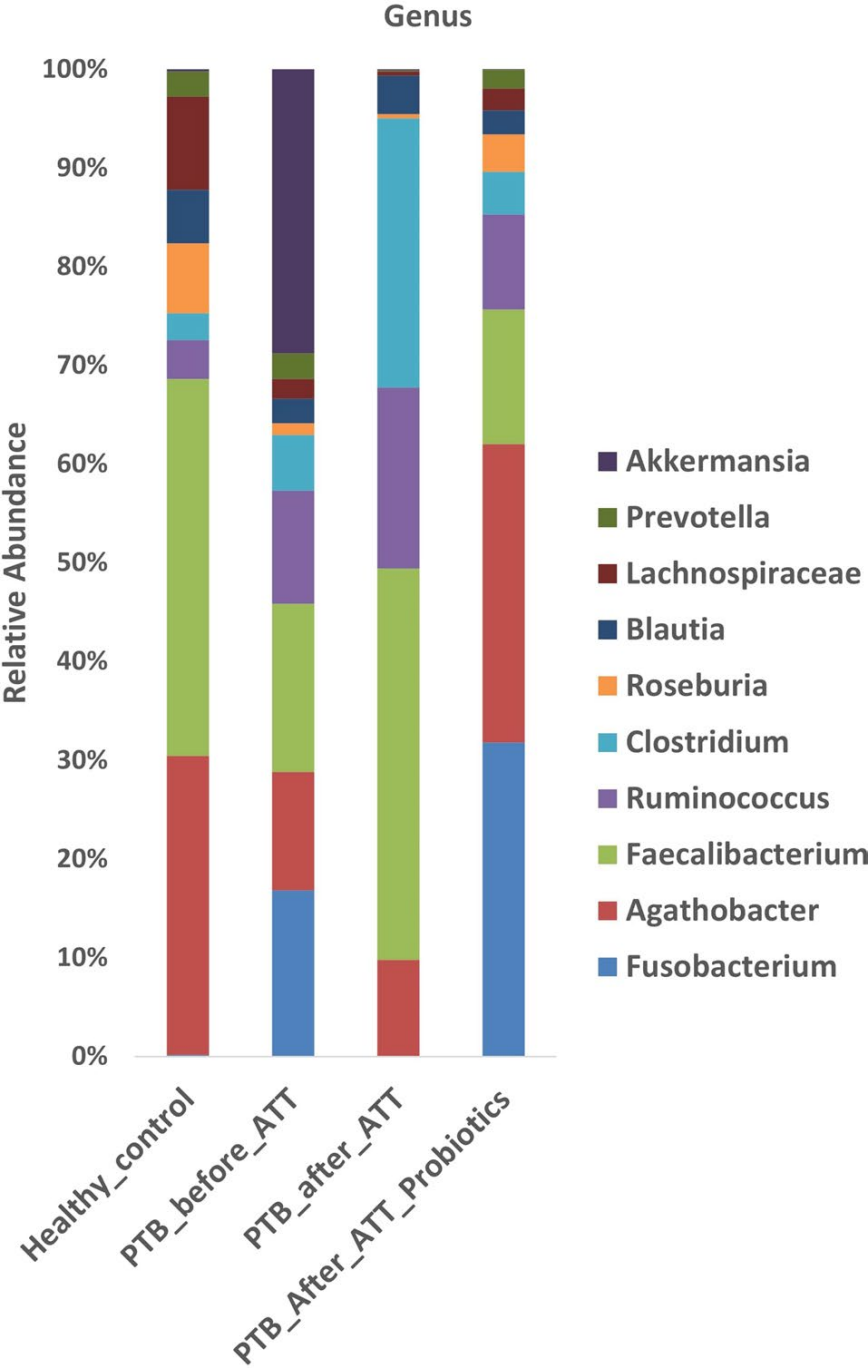
Relative abundance at genus level

The identified ASVs were categorized at the phylum and genus levels to determine gut microbiota composition, focusing on the top ten most abundant taxa (Figs. 1 and 2). At the taxa level, gut microbiota composition exhibited marked differences across the four groups (HC, PTB_before_ATT, PTB_after_ATT, and PTB_after_ATT_Probiotics). Firmicutes were the dominant phylum in all groups. In the HC group, Firmicutes constituted most of the microbial community, followed by Bacteroidota, Cyanobacteria, Actinobacteriota, Proteobacteria, Campylobacteriota, etc. At the genus level in the HC group, the most abundant genera included *Faecalibacterium*, *Agathobacter*, *Lachnospiraceae*,* and Roseburia*, followed by a lesser proportion of *Blautia*,* Ruminococcus*,* Prevotella*, and *Clostridium.* In the PTB_before_ATT group, the proportion of phylum Firmicutes was lower compared to the HC group, with the increased relative abundance of Verrucomicrobiota, Proteobacteria, Fusobacteriota, Actinobacteriota, Bacteroidota, etc. At the genus level, the PTB_before_ATT group, *Akkermansia*, was the most predominant genus, followed by *Faecalibacterium*,* Fusobacterium*,* Agathobacter*,* Ruminococcus*,* and Clostridium*. Other genera, such as *Prevotella*,* Blautia*,* Lachnospiraceae*, and *Roseburia*, were in smaller proportions. In PTB_after_ATT, Firmicutes remained predominant, followed by a significant proportion of Proteobacteria, Bacteroida, and Actinobacteriota. In PTB_after_ATT_Probiotics, a considerable proportion of phyla were Firmicutes, Fusobacteriota, Bacteroidota, Proteobacteria, and Actinobacteriota. At the genus level, dominant genera were in order of *Faecalibacterium*, *Clostridium*,* Ruminococcus*,* Agathobacter*, and *Blautia.* In the PTB_after_ATT_Probiotics group, the dominant genera were *Agathobacter*,* Fusobacterium*,* Faecalibacterium*,* Ruminococcus*,* Clostridium*, and *Roseburia*.

### Gut microbiota diversity

We conducted microbial diversity analysis in PTB_before_ATT (*n* = 18), PTB_after_ATT (*n* = 4), PTB_after_ATT Probiotics (*n* = 5) with respect to HC (*n* = 12).

**Fig. 3:**
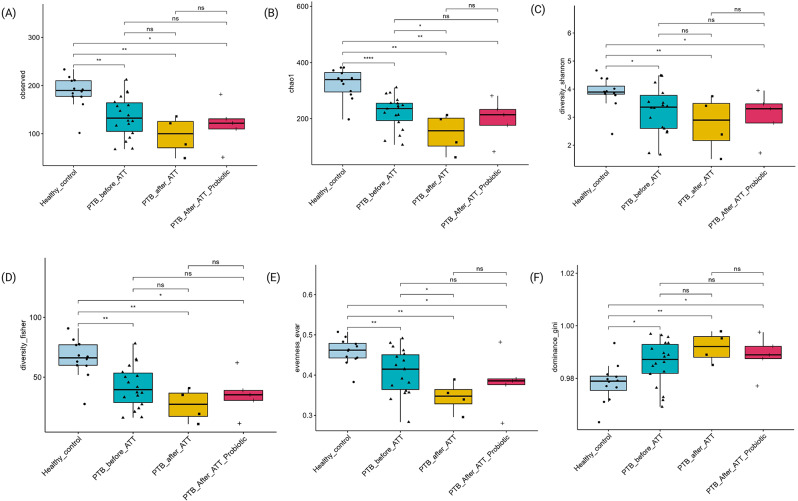
Alpha diversity among all groups. *Note*. HC (*n* = 12); PTB_before_ATT (*n* = 18), 2 out of 20 excluded after rarefaction; PTB_after_ATT (*n* = 4); PTB_after_ATT_Probiotics (*n* = 5). ns: *p* > 0.05; *: *p* ≤ 0.05; **: *p* ≤ 0.01; ***: *p* ≤ 0.001; ****: ≤ 0.0001. (A) Observed taxa, (B)Chao1, (C) Shannon diversity, (D) Fisher diversity, (E) Evenness evar, (F) Dominance gini

We evaluated alpha diversity across all groups using multiple metrics, including observed taxa, Chao1, Shannon diversity, Fisher diversity, Evenness evar, and dominance Gini index (Fig. 3). From the observed taxa, Chao1, Shannon diversity, and Fisher diversity, we observed that the microbial community richness and diversity were significantly depleted in PTB patients before ATT initiation than in HC (Fig. 3).

**Fig. 4:**
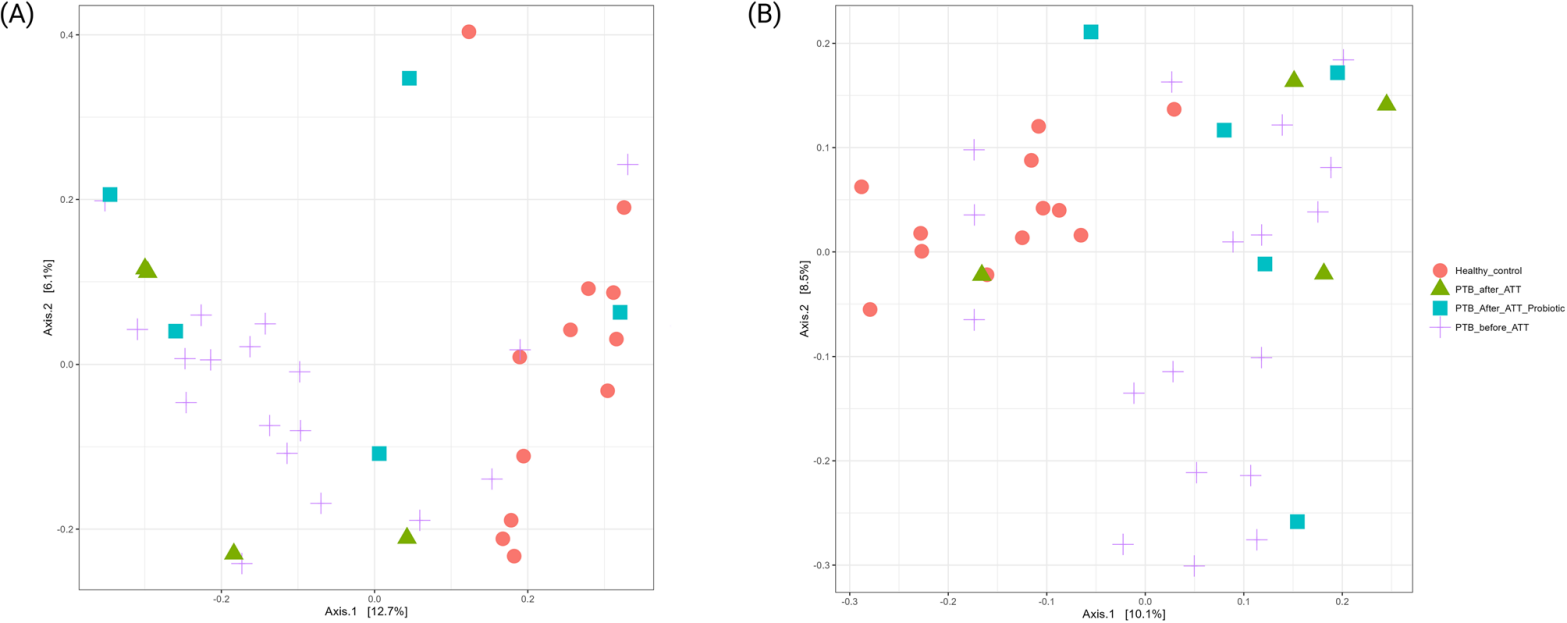
Beta diversity among all groups. *Note*. HC (*n* = 12); PTB_before_ATT (*n* = 18), 2 out of 20 excluded after rarefaction; PTB_after_ATT (*n* = 4); PTB_after_ATT_Probiotics (*n* = 5). (A) Bray Curtis dissimilarity, (B) Weighted UniFrac distances

Beta diversity was assessed using Bray-Curtis dissimilarity and weighted UniFrac distances (Fig. 4). In the pairwise comparison, significant differences were observed in both Bray-Curtis dissimilarity and weighted UniFrac distances (*p* < 0.05), indicating notable differences in the microbial community composition between the groups. Significant differences were observed between HC and the following groups: PTB_before_ATT (*p* = 0.001), PTB_after_ATT (*p* = 0.004), and PTB_after_ATT_Probiotics (*p* = 0.003) for Bray-Curtis dissimilarity, and HC vs. PTB_before_ATT (*p* = 0.001), HC vs. PTB_after_ATT (*p* = 0.002), and HC vs. PTB_after_ATT_Probiotics (*p* = 0.007) for weighted UniFrac. PCoA plots were generated to visualize the beta diversity patterns, which supported the PERMANOVA results, clearly illustrating the distinct clustering of the groups relative to HC.

The significant dissimilarities in microbial composition between HCs and PTB groups (before ATT, after ATT, and after ATT with probiotics) suggest the presence of a PTB-associated microbial signature. This likely reflects a dysbiotic state characterized by microbial diversity, richness, and composition alterations. Beta diversity metrics imply that even with ATT, the microbial community structure in PTB patients remained distinct from that of HCs. This suggests that gut dysbiosis remains resilient, as the PTB-associated microbial signature persists even after conventional treatment.

To explore within-group changes, we performed subgroup analyses. No significant differences were detected in either alpha diversity or beta diversity metrics in the group of PTB patients with paired samples before and after ATT (Supplementary file 3; Figure S1 & S2). However, in the subgroup analysis for paired samples before and after ATT receiving probiotics (*n* = 5), significant differences were observed in richness (Chao1, observed taxa), diversity (Fisher diversity), and the rarity of low-abundance taxa (Supplementary file 3; Figure S3). While these findings point to probiotics’ targeted modulation of the microbial signature, beta diversity metrics did not show significant shifts (Supplementary file 3; Figure S4). Probiotics alongside ATT may influence specific gut microbiota components without altering the more comprehensive PTB-associated microbial signature.

### Differential abundance analysis

**Fig. 5:**
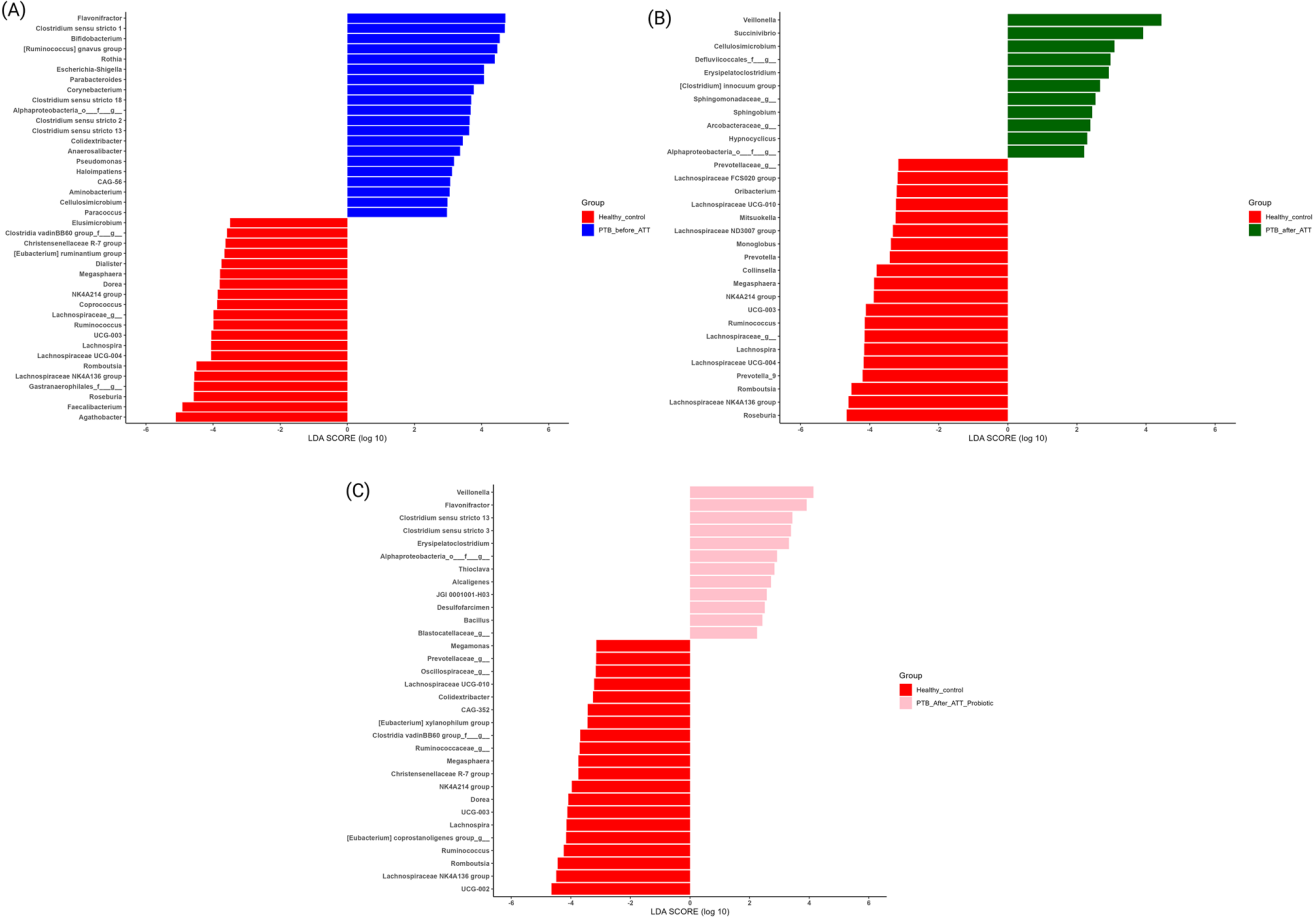
LEfSe analysis. *Note*. LEfSe Analysis at aggregate genus level. (A) HC (*n* = 12) Vs. PTB_before_ATT (*n* = 20), (B) HC (*n* = 12) Vs. PTB_after_ATT (*n* = 4), (C) HC (*n* = 12) Vs. PTB_after_ATT_Probiotics (*n* = 5)

We conducted the LEfSe analysis in PTB before ATT (*n* = 20), PTB after ATT (*n* = 4), PTB after ATT Probiotics (*n* = 5) with respect to HC (*n* = 12) to identify the marker taxa in the influence of PTB disease, ATT, and ATT with Probiotics on the gut microbial community. At the genus level, 40 discriminating genera were detected for HC Vs. PTB before the ATT group, of which 20 genera belong to PTB before the ATT group (Fig. 5A). Some of the significant marker genera in the HC group were *Agathobacter*,* Facecaibacterium*,* Roseburia*,* Lachnospiraceae NK4A136 group*,* Romboutsia*,* Lachnospira*,* Ruminococcus*,* Coprococcus*, and *Dorea*. Whereas in PTB before the ATT group, some of the significant marker genera were *Flavonifractor*,* Clostridium sensu stricto 1*,* Bifidobacterium*,* Rothia*,* Parabacteroides*,* Corynebacterium*,* Clostridium sensu stricto 18*,* Clostridium sensu stricto 2*, and *Clostridium sensu stricto 13*. From LEfSe analysis in HC Vs. PTB after ATT group, we identified 11 marker genera (Fig. 5B) in PTB after ATT group. Some of which are *Veillonella*,* Succinivibrio*,* Cellulosimicrobium*,* Erysipelatoclostridium*,* [Clostridium]innocuum group*,* Sphingobium*,* Hypnocyclicus*, and *Alphaproteobacteria*. In PTB_after_ATT_Probiotic group, we detected the 12 marker genera (Fig. 5C). Some of which are *Veillonella*,* Flavonifractor*,* Clostridium sensu stricto 13*,* Clostridium sensu stricto 3*,* Erysipelatoclostridium*,* Alphaproteobacteria*,* Thioclava*,* Alcaligenes*,* JGI 0001001-H03*,* Desulfofarcimen*, and *Bacillus.* We identified four marker genera each from the paired samples comparison of PTB patients before and after receiving ATT, and before and after receiving ATT with probiotics, respectively (Supplementary file 3; Figure S5).

### Predicted functional analysis

Microbial metagenomic inference was performed using PICRUSt2, and bar plots were generated for significant pathways (*p* < 0.05) based on ALDEx2.

**Fig. 6:**
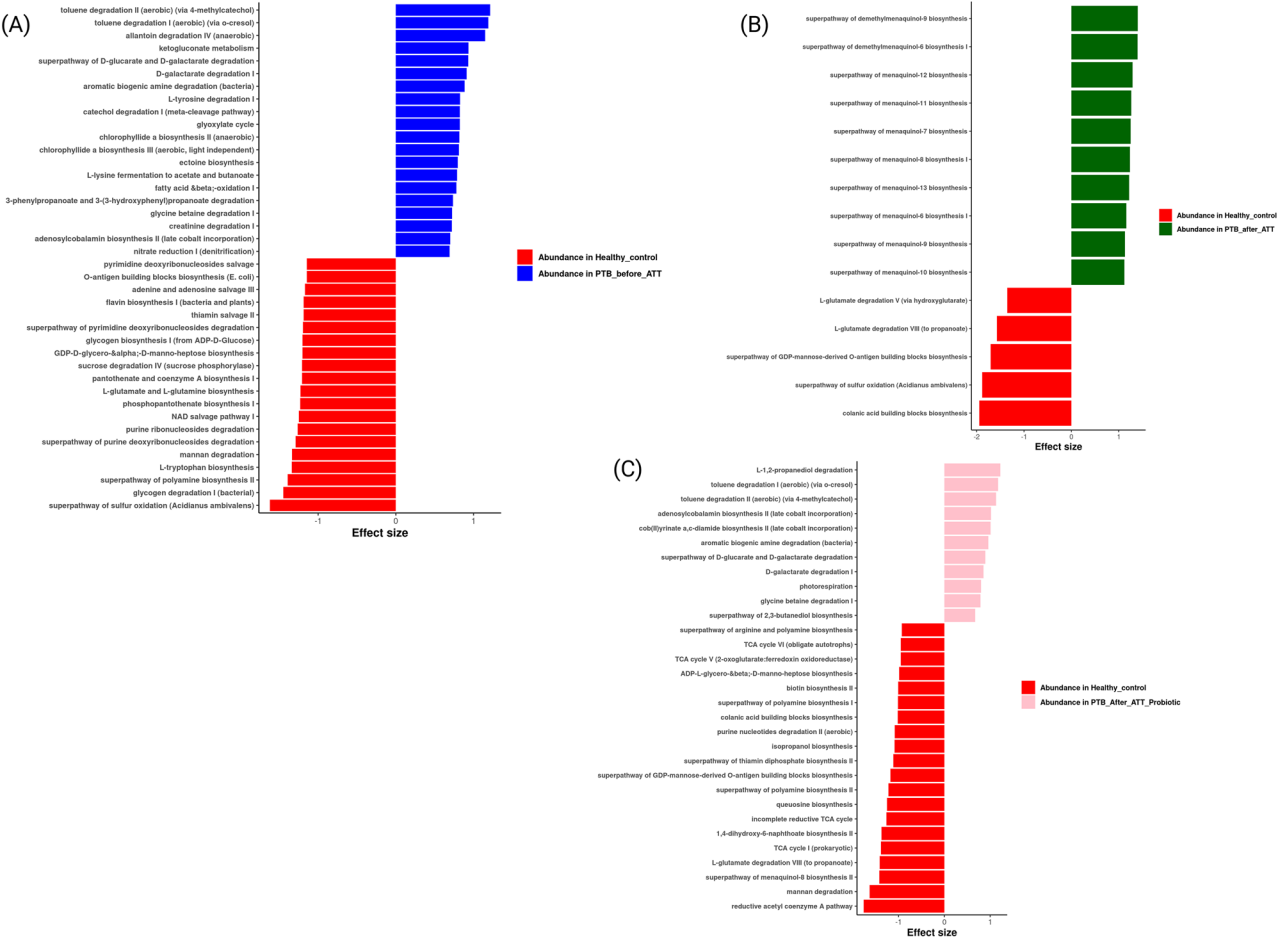
PICRUSt2 analysis *Note*. (A) HC (*n* = 12) Vs. PTB_before_ATT (*n* = 20), (B) HC (*n* = 12) Vs. PTB_after_ATT (*n* = 4), (C) HC (*n* = 12) Vs. PTB_after_ATT_Probiotics (*n* = 5)

We identified 40 active features in HC Vs. PTB_before_ATT group (Fig. 6A). Pathways related mostly to biosynthesis and salvage of key biomolecules were abundant in the HC group. Some of them were the super pathway of sulfur oxidation (Acidianus ambivalens), glycogen degradation I (bacterial), super pathway of polyamine biosynthesis II, L-tryptophan biosynthesis, purine ribonucleosides salvage, NAD salvage pathway I, pantothenate and coenzyme A biosynthesis I, phosphopantothenate biosynthesis I, glycogen biosynthesis I (from ADP-D-glucose), flavin biosynthesis I (bacteria and plants), and thiamin salvage II. In the PTB_before_ATT group, pathways mainly related to degradation metabolic processes were identified to be abundant (Fig. 6A). Some of the pathways were toluene degradation II (aerobic) (via 4-methylcatechol), toluene degradation I (aerobic) (via o-cresol), allantoin degradation IV (anaerobic), ketogluconate metabolism, superpathway of D-glucarate and D-galactarate degradation, D-galactarate degradation I, aromatic biogenic amine degradation (catabolism), L-tyrosine degradation I, catechol degradation I (meta-cleavage pathway), and glyoxylate cycle.

We performed a pairwise comparison of HC Vs. PTB_after_ATT to assess the impact of ATT in PTB patients, which resulted in 15 active features (Fig. 6B). Several pathways related to menaquinone biosynthesis (Vitamin K2) showed significant enrichment in the PTB_after_ATT group.

To infer the impact of probiotics alongside ATT in PTB patients, we have performed a comparison between HC Vs. PTB_after_ATT_Probiotics, which resulted in 31 active features (Fig. 6C). L-1,2-propanediol degradation, tolulene degradation I (aerobic) (via o-cresol), tolulene degradation II (aerobic) (via 4-methylcatechol), adenosylcobalamin biosynthesis II (late cobalt incorporation), cob(II)yrinate a, c-diamide biosynthesis II (late cobalt incorporation), aromatic biogenic amine degradation (bacteria), superpathway of D-glucarate and D-galactarate degradation, D-galactarate degradation I, photorespiration, glycine betaine degradation I, and superpathway of 2,3-butanediol biosynthesis pathways were abundant in the PTB_after_ATT_Probiotics group. The before and after comparison for ATT and ATT_probiotics identified 13 and 6 active features, respectively (Supplementary file 3; Figure S6).

## Discussion

This study provides insights into the gut microbial changes linked to PTB within the southern Indian demographic. The diversity of gut bacterial microbiota decreased more in the naïve PTB patient group than in the HC group. Furthermore, beta diversity demonstrated notable differences in microbial community composition between PTB patients (PTB_before_ATT) and HC (*p* = 0.001), as evidenced by distinct clustering in PCoA plots. These findings are corroborating earlier research [17, 18]. *Ding et al. 2022* observed a significant reduction in gut microbial diversity among PTB patients compared to HC [17]. Similarly, *Wang et al. 2021* reported distinct clustering patterns in PTB patients, reflecting substantial shifts in the microbial community structure [18].

Our study findings from comparing the PTB_after_ATT group with the HC group revealed that the conventional treatment by ATT further exacerbates microbial dysbiosis in PTB patients than in naïve PTB patients. This suggests that while ATT is essential for managing PTB, it may worsen gut microbial imbalances. The resilience of dysbiosis, as evidenced by persistent differences from HCs, underscores the need for adjunctive interventions to restore microbial health during treatment. Notably, in our study, samples from PTB patients were collected after the ATT exposure time was limited to two months (intensive phase). This indicates that ATT quickly and substantially affects gut microbial depletion. *Wipperman et al. 2017* demonstrated that dysbiosis induced by ATT persists for at least 14 months, implying ATT’s long-term impact on the gut microbial community [19]. Similarly, *Séraphin et al. 2023* concluded that rifamycin-based TB preventive therapy also induces depletion in microbial diversity [20]. The alpha diversity indices increased two months after rifamycin treatment, yet failed to achieve the baseline level, implying the incomplete recovery in microbial community diversity. Furthermore, the potential impact of mono and combined drug therapy for TB patients could be considered, as different drug regimens may have varying effects on the degree of gut microbiota dysbiosis.

The subgroup analysis from paired samples comparison of PTB patients before and after receiving ATT did not yield any significance in microbial diversity, yet showed marker taxa in differential abundance analysis. Nevertheless, it is crucial to recognize that our findings result from a minimal sample size of PTB patients after ATT initiation, which restricts the statistical generalizability of these findings. Despite this limitation, we observed significant changes in taxa from differential abundance analysis at the genus level, emphasizing specific microbial modifications linked to ATT.

Interestingly, the supplementation of probiotics alongside ATT demonstrated a modest but notable improvement in microbial diversity. In the PTB_after_ATT_Probiotics group, alpha diversity metrics showed mild increases compared to the PTB_after_ATT group. However, this improvement was not sufficient to achieve statistical equivalence with HCs. Yet, the observed trends suggest a beneficial modulation of gut microbiota by probiotics. Probiotic supplementation during ATT is likely to modulate the disruption of the gut microbiota, which ATT alone does not address. It emphasizes how probiotics may maintain or recover microbial diversity in PTB patients during the long-term ATT. *John et al. 2024* concluded that the probiotic may minimize the disruption of antibiotic treatment on the gut microbiome by preserving microbial diversity and even reducing the abundance of antimicrobial resistance genes [21]. Subgroup analyses provided further insights into the role of probiotics. Paired sample comparisons of PTB patients before and after ATT, receiving probiotics, revealed significant improvements in alpha diversity metrics (Chao1, Observed taxa, Fisher diversity, rarity of low-abundance taxa). However, beta diversity metrics in these paired before and after analyses did not exhibit significant shifts, indicating that probiotics may influence specific microbial components rather than driving broader compositional changes. It is important to note that in this before-and-after ATT probiotics pairwise comparison, rarefaction was not performed before diversity analysis to retain all matched-pairs samples. While this approach preserves within-sample pairing for probiotics supplementation, it may limit the direct comparability of diversity metrics with other rarefied group comparisons.

Healthy human gut microbiota is dominated by the phyla Firmicutes and Bacteroidota [22], consistent with our findings in the HC group. Both active PTB disease and ATT are associated with gut microbiota dysbiosis [23–25]. Similarly, our study findings revealed differences in gut microbiota composition in PTB_before_ATT and PTB_after_ATT with respect to HC, suggesting a dysbiotic state.

Our study findings identified distinct microbial signatures across groups. In comparisons between HC and PTB_before_ATT, including *Faecalibacterium*,* Roseburia*,* Agathobacter*,* Coprococcus*,* Ruminococcus*, and *Lachnospiraceae NK4A136 group*, were significantly deregulated in PTB_before_ATT. These genera are known as Short-Chain Fatty Acids (SCFAs) producers [26–31]. SCFAs, specifically acetate, propionate, and butyrate, are the primary metabolites generated by the gut microbiota. SCFAs have a variety of functions such as anti-inflammatory, immune regulations, etc [32]. Therefore, the depletion of SCFAs-producing gut commensals may contribute to immune dysregulation and systemic inflammation in PTB.

The genus *Erysipelatoclostridium* was abundant during the ATT, as reported by Wipperman et al., 2017, and Hu et al., 2019 [19, 33]. *Erysipelatoclostridium* is enriched during inflammatory and immune-reducing conditions, such as in TB patients [34]. These findings are consistent with our study findings, as we observed the following ATT in the PTB_after_ATT group. Furthermore, we detected the genus *Erysipelatoclostridium* in the PTB_after_ATT_Probiotics group, suggesting that this genus has persisted even with short-term probiotics supplementation. As per Wipperman et al. 2017 [19], the genus *Erysipelatoclostridium* persisted for three months following ATT. This indicates a long-term microbial shift induced by treatment. A short-term probiotics supplementation in our study may have been insufficient to restore microbial equilibrium or eliminate persistent taxa such as *Erysipelatoclostridium*. The marker genus *Erysipelatoclostridium* suggests continued dysbiosis or a transitional microbial state.

The marker genera, such as *Clostridium sensu stricto 2*,* Terrisporobacter*,* Eubacterium*, and *Paraclostridium*, were identified from paired samples comparison of PTB patients before and after receiving ATT. Furthermore, these genera are butyrate producers, which were identified to be depleted post-ATT [35–38], similar to our study findings. Genus *Clostridium sensu stricto* is generally considered the true *Clostridium* genus [39]. Some species within *Clostridium sensu stricto* can be pathogenic, while others are considered beneficial. However, most species are considered harmless and responsible for SCFAs production [39]. These *Clostridium sensu stricto* species, as marker genera for microbial health, could be valuable targets for future studies.

In this study, microbial metagenomic inference was conducted to predict functional pathways that distinguish HC from PTB patients. Our findings revealed a deregulation of functional pathways in PTB patients following ATT, suggesting shifts in gut microbiota function associated with the treatment. However, probiotics supplementation during ATT resulted in more active metabolic pathways than ATT alone. Pathways enriched in HC are primarily involved in biosynthesis and energy metabolism, suggesting that healthy individuals’ gut microbiota is functionally balanced, supporting normal gut and host metabolism. The inferred pathways in PTB_before_ATT patients indicate significant dysbiosis with a shift towards catabolic and degradation pathways, which could be adapting to a pathological or nutrient-deprived environment. Menaquinone (Vitamin K2) biosynthesis is crucial in bacterial respiration, particularly in Gram-positive bacteria. Certain bacteria, including those in the gut, rely on menaquinone for electron transport in aerobic or anaerobic respiration. Respiration occurs in the cell membrane of prokaryotic cells [40]. An increase in Menaquinone biosynthesis pathways in PTB_after_ATT patients could indicate a microbial shift towards metabolic pathways that support native gut commensal bacterial survival and energy production in response to production imbalance during the treatment period. The before- and -after comparison of ATT_probiotics revealed enrichment in the L-methionine biosynthesis pathway, indicating the possible influence on gut microbiota functionality due to probiotics supplementation during ATT.

The current commercially available probiotics supplements can provide metabolically and ecologically beneficial effects, implying dynamic effects. These are not meant for gut colonization. These commercially available probiotic strains tend to pass through the digestive system and are usually flushed out within a few days [4]. For TB patients undergoing ATT, which has a minimum duration of six months, supplementation with probiotics for just one or two months may have a limited impact. Probiotic supplementation may not dynamically change the gut microbial community by displacing the native commensal microbes. Instead, it may only maintain the gut microbiota stability for eubiosis, protecting from the exacerbation of dysbiosis during ATT.

Our study only employed a 16 S amplicon sequencing approach for studying metataxonomics, which gave us the landscape of microbial community diversity and taxonomy. Future studies may focus on integrative multi-omics approaches such as metagenomics to capture the functional gene repertoire, metatranscriptomics to understand microbial transcriptional activity, and metabolomics to profile the metabolite outputs of microbial communities. Such approaches would facilitate a more profound exploration of the development of precision microbiome-based interventions.

Conventional probiotics are designed to benefit overall gut health. They are used for common issues like digestive discomfort, immune support, or general microbiome balance in the “one size fits all” concept. However, next-generation probiotics, defined as “live microorganisms identified based on comparative microbiota analyses that, when administered in adequate amounts, confer a health benefit on the host”, are more promising, with the potential for customized probiotic treatments [41, 42]. With a precision therapeutic approach, interventions like next-generation probiotics and prebiotics can be tailored to the local environment and the microbiome traits of a person or population [41, 42].

The follow-up duration in our study was limited to less than two months, which may not be sufficient to fully capture the long-term dynamics of the gut microbiota in PTB patients. Additionally, the relatively small sample size, particularly in the longitudinal comparison for subgroup analysis (*n* = 4 for PTB_after_ATT and *n* = 5 for PTB_After_ATT_Probiotic groups, respectively), substantially limits the statistical power and generalizability of the findings. Furthermore, we did not collect detailed information on participants’ diet or lifestyle factors, which could have significantly influenced gut microbiota composition. The absence of this data limits the ability to fully contextualize the observed microbial changes. While our study findings provide initial insights into the potential influence of probiotics, further studies with larger cohorts and extended follow-up are required to validate these results. Such studies are also needed to characterize the long-term impacts of ATT and probiotics on gut microbial communities, especially those taxes involved in SCFA production.

## Conclusions

The study identified distinct gut microbial features in PTB patients in the south Indian population. The gut microbial diversity depletion was found among PTB patients, and this was further exacerbated during ATT. The supplementation of probiotics alongside ATT showed a trend toward restoring microbial diversity to higher levels than ATT alone. However, this improvement did not reach statistical significance. Moreover, our analysis revealed a notable depletion in the predicted metabolic pathways in PTB patients undergoing ATT, with biosynthesis pathways mainly affected. Furthermore, the study identified distinct microbial taxa in PTB patients before ATT initiation and during ATT, which could serve as potential biomarkers, highlighting opportunities for future research to explore targeted microbiome-based interventions to mitigate treatment-associated dysbiosis. Nevertheless, these findings warrant careful understanding due to the very small sample size in the follow-up groups, which limits generalizability. Larger, well-controlled studies are needed to validate and expand upon these preliminary results.

## Supplementary Information

Below is the link to the electronic supplementary material.

Supplementary Material 1

Supplementary Material 2

Supplementary Material 3

## Data Availability

The 16 S sequence reads have been deposited under NCBI BioProject number PRJNA1207258.

## Abbreviations

ALDEx2: ANOVA-like differential expression
ASVs: Amplicon Sequence Variants
ATT: Antitubercular therapy
DADA2: Divisive Amplicon Denoising Algorithm 2
HC: Healthy control
LDA: Linear Discriminant Analysis
LEfSe: Linear Discriminant Analysis Effect Size
Mtb: Mycobacterium tuberculosis
PCoA: Principal Coordinate Analysis
PERMANOVA: permutational multivariate analysis of variance
PICRUSt2: Phylogenetic investigation of communities by reconstruction of unobserved states 2.0
PTB: Pulmonary tuberculosis
SCFAs: Short-Chain Fatty Acids
TB: Tuberculosis
UniFrac: Unique Fraction Metric
