# Supplementary material for: Gut microbiota profile in newly diagnosed pulmonary tuberculosis patients: an exploratory pilot study in southern India
Source: Gut Pathog. 2025 Aug 11;17:59. doi: 10.1186/s13099-025-00736-x (PMC12337371; doi:10.1186/s13099-025-00736-x)
Supplement: Supplementary file 1 — Supplementary Material 1 [file 13099_2025_736_MOESM1_ESM.docx]

**Table S1: Demographics of enrolled participants**

| Characteristics | PTB patients  (n=20) | HC  (n=12) |
| --- | --- | --- |
| Age (years) (Mean) | 44.4 | 40.75 |
| Gender  Male  Female | 15  05 | 09  03 |
| Diabetes status  Yes  No | 08  12 | 0  12 |

PTB: Pulmonary tuberculosis; HC: Healthy control

**Figure S1: Flow diagram of participant enrollment and group allocation**


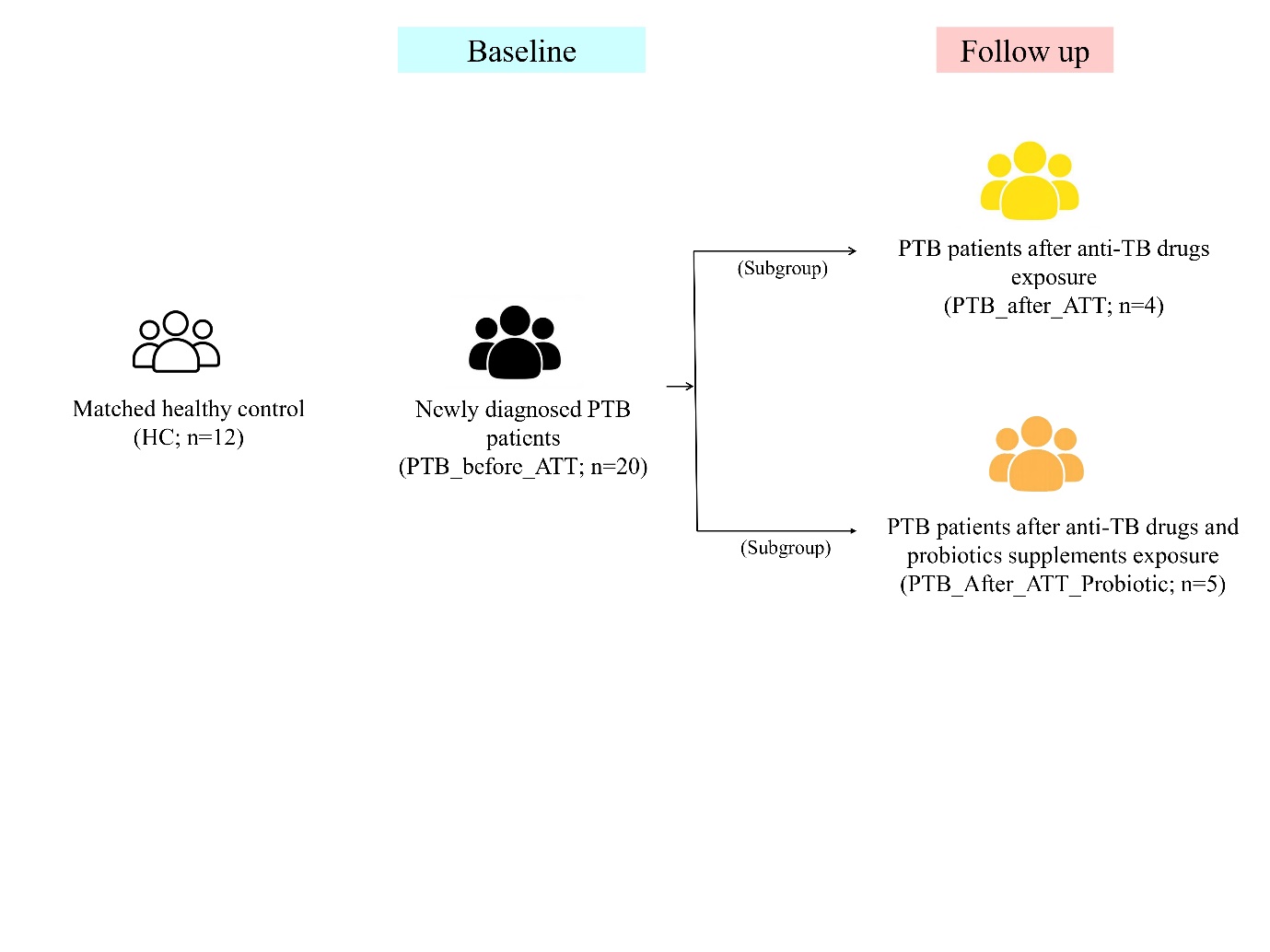


TB: Tuberculosis; PTB: Pulmonary tuberculosis; ATT: Antitubercular therapy

**Figure S2: Rarefaction curve of sequencing data from 41 fecal samples before normalization**


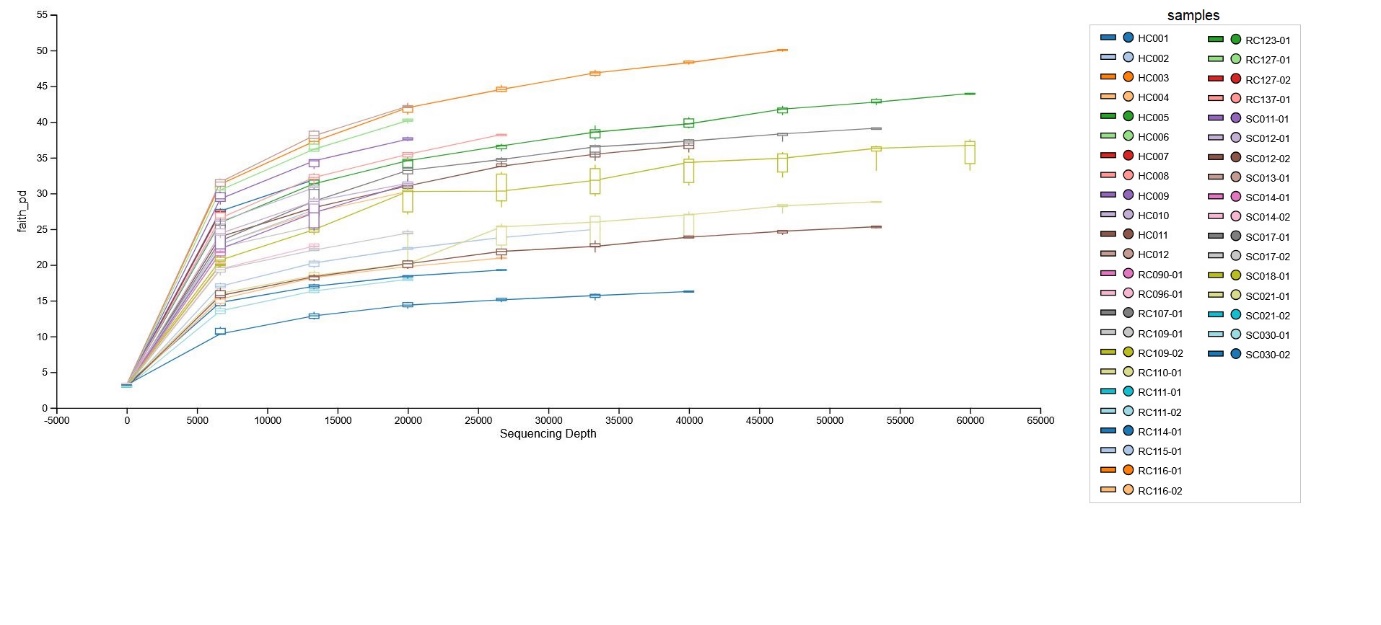


Samples with fewer than 1103 sequences were excluded for alpha and beta diversity analysis.

**Figure S3: Rarefaction curve of sequencing data from 41 fecal samples after normalization**

**
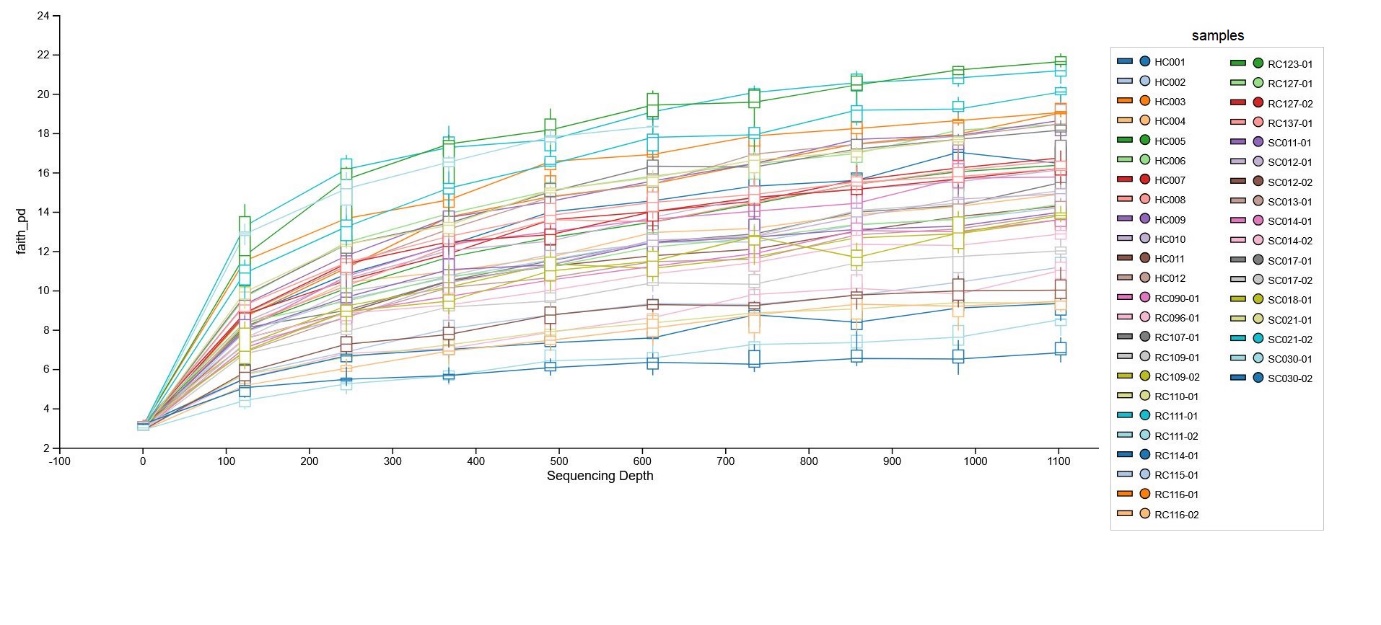
**
