# Supplementary material for: Gut microbiota profile in newly diagnosed pulmonary tuberculosis patients: an exploratory pilot study in southern India
Source: Gut Pathog. 2025 Aug 11;17:59. doi: 10.1186/s13099-025-00736-x (PMC12337371; doi:10.1186/s13099-025-00736-x)
Supplement: Supplementary file 3 — Supplementary Material 3 [file 13099_2025_736_MOESM3_ESM.docx]

**Diversity analysis for the subgroup analyses for the before and after paired samples**

**Alpha and beta diversity for the subgroup analyses for the paired samples before and after ATT (n=4)**

**
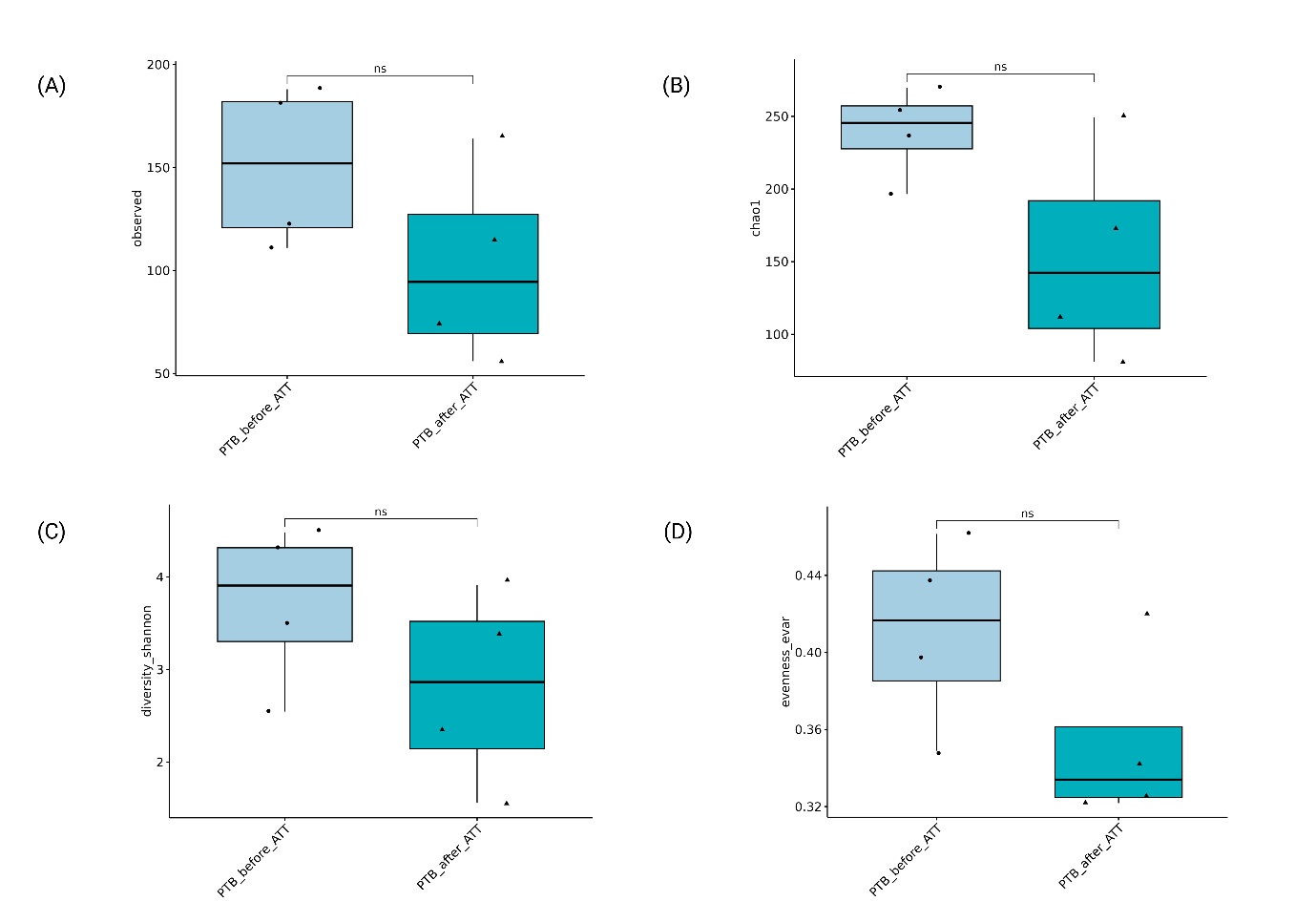
**

**Figure S 1: Alpha Diversity.** ns: p > 0.05; *: p <= 0.05; **: p <= 0.01; ***: p <= 0.001; ****: p <= 0.0001. (A) Observed taxa, (B) Chao1, (C) Shannon diversity, (D) Evenness evar


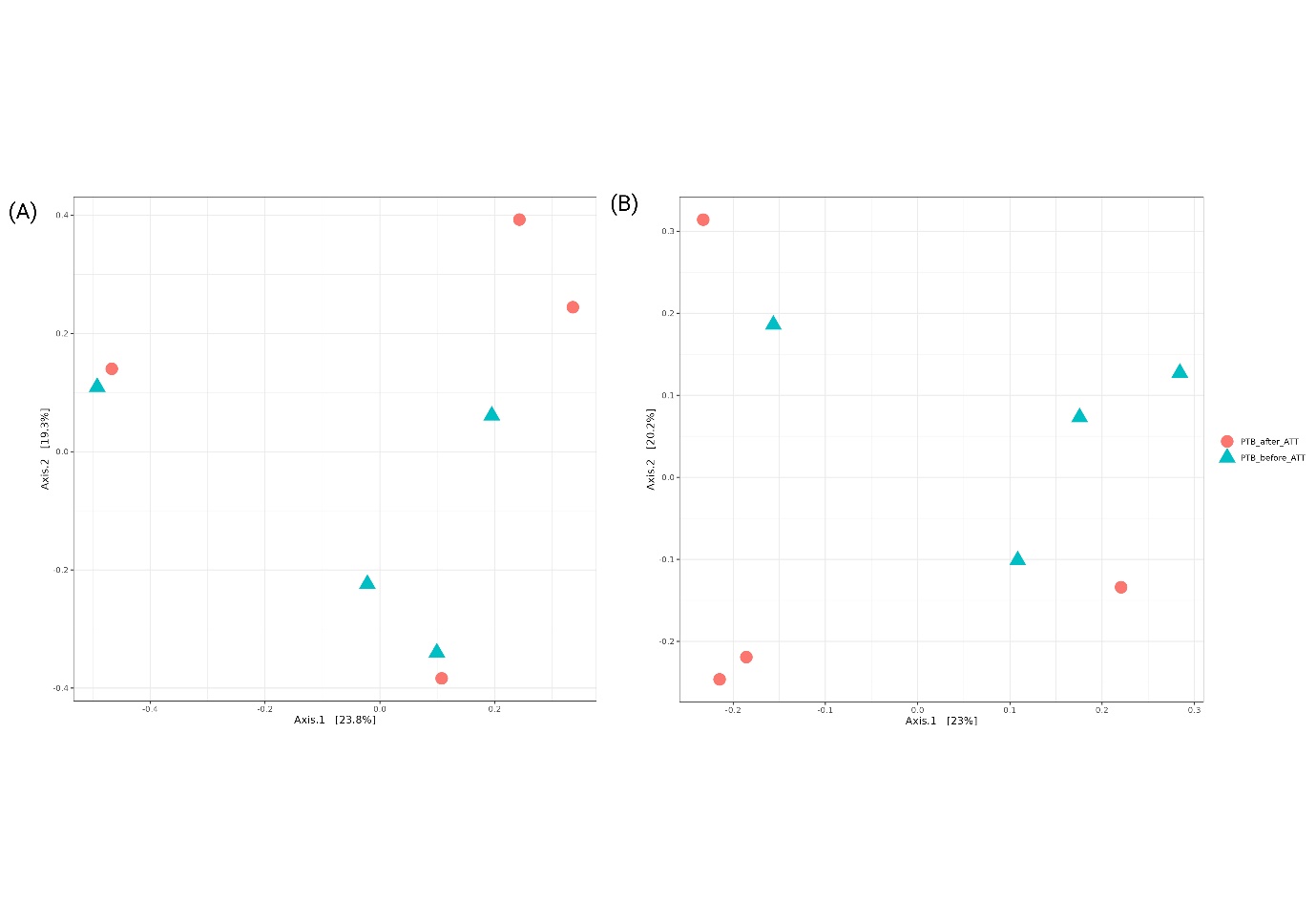


**Figure S 2: Beta diversity. (**A) Bray curtis dissimilarity, (B) Weighted UniFrac distances

**Alpha and beta diversity for the subgroup analyses for the paired samples before and ATT_Probiotics (n=5)**

**
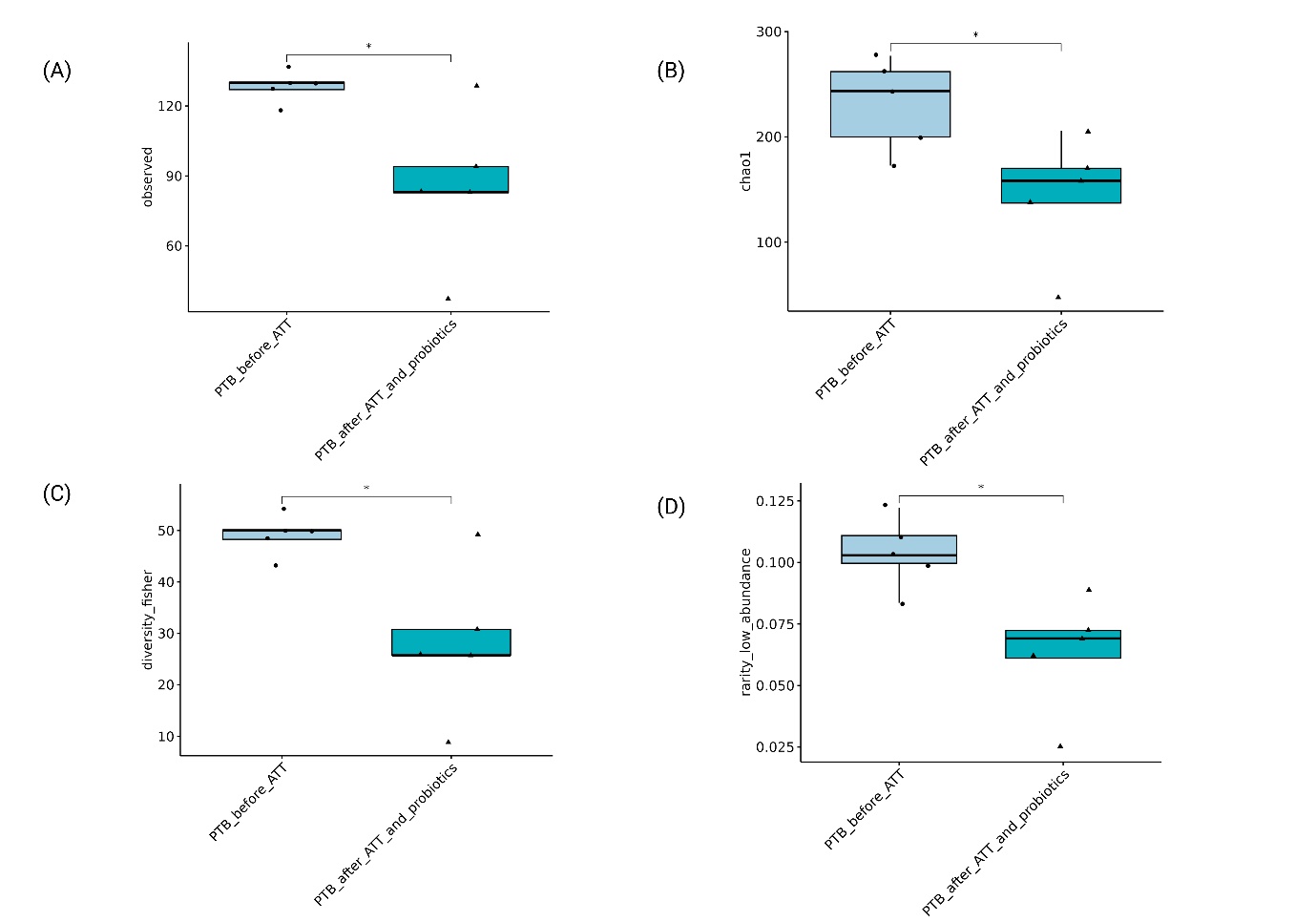
**

**Figure S 3: Alpha Diversity.** ns: p > 0.05; *: p <= 0.05; **: p <= 0.01; ***: p <= 0.001; ****: p <= 0.0001. A) Observed taxa, (B) Chao1, (C) Fisher diversity, (D) Rarity low abundance. *Note: Rarefaction was not performed in this analysis*


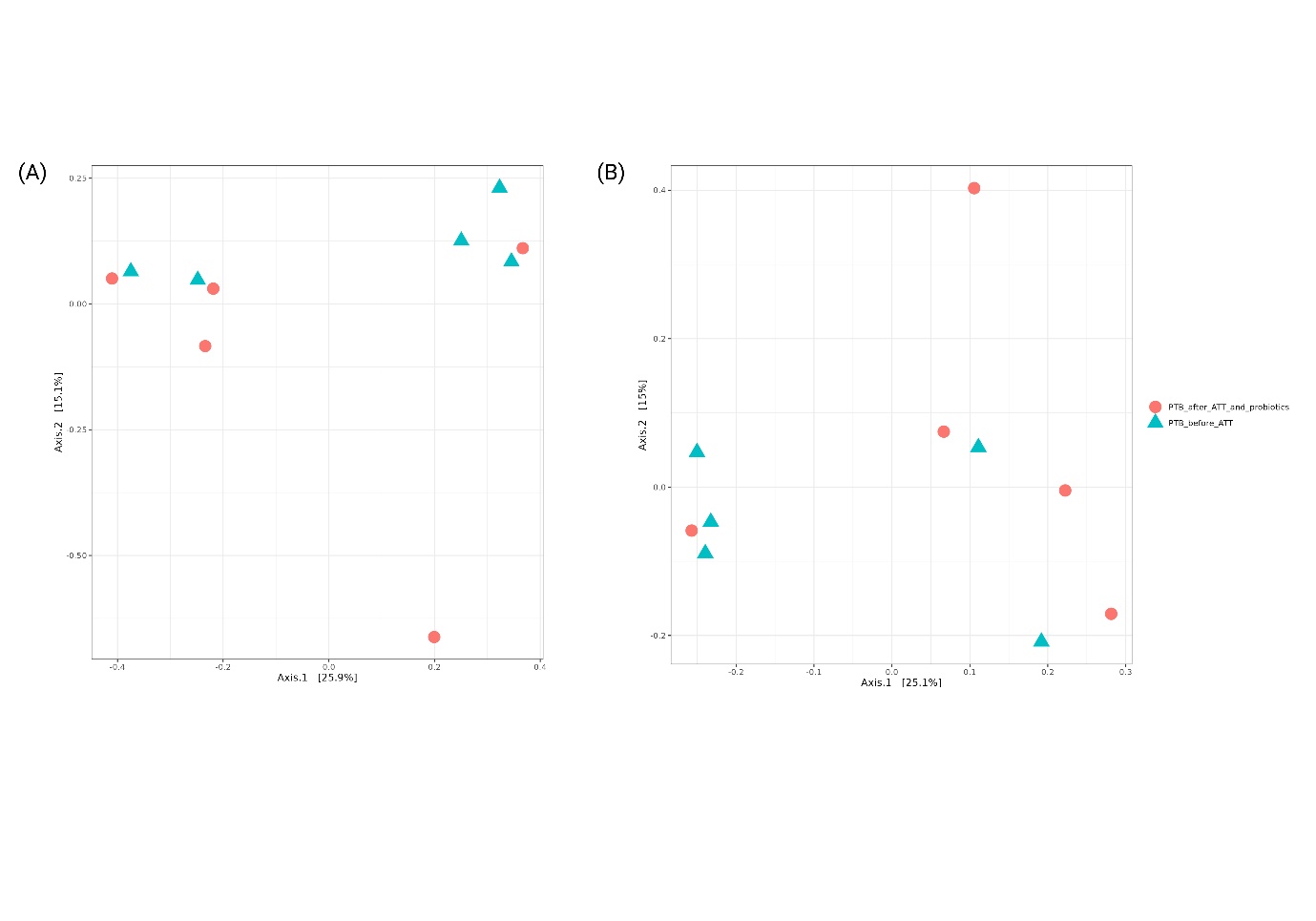


**Figure S 4: Beta diversity. (**A) Bray curtis dissimilarity, (B) Weighted UniFrac distances. *Note: Rarefaction was not performed in this analysis*

**Differential abundance analysis for the subgroup analyses for the before and after paired samples**

**
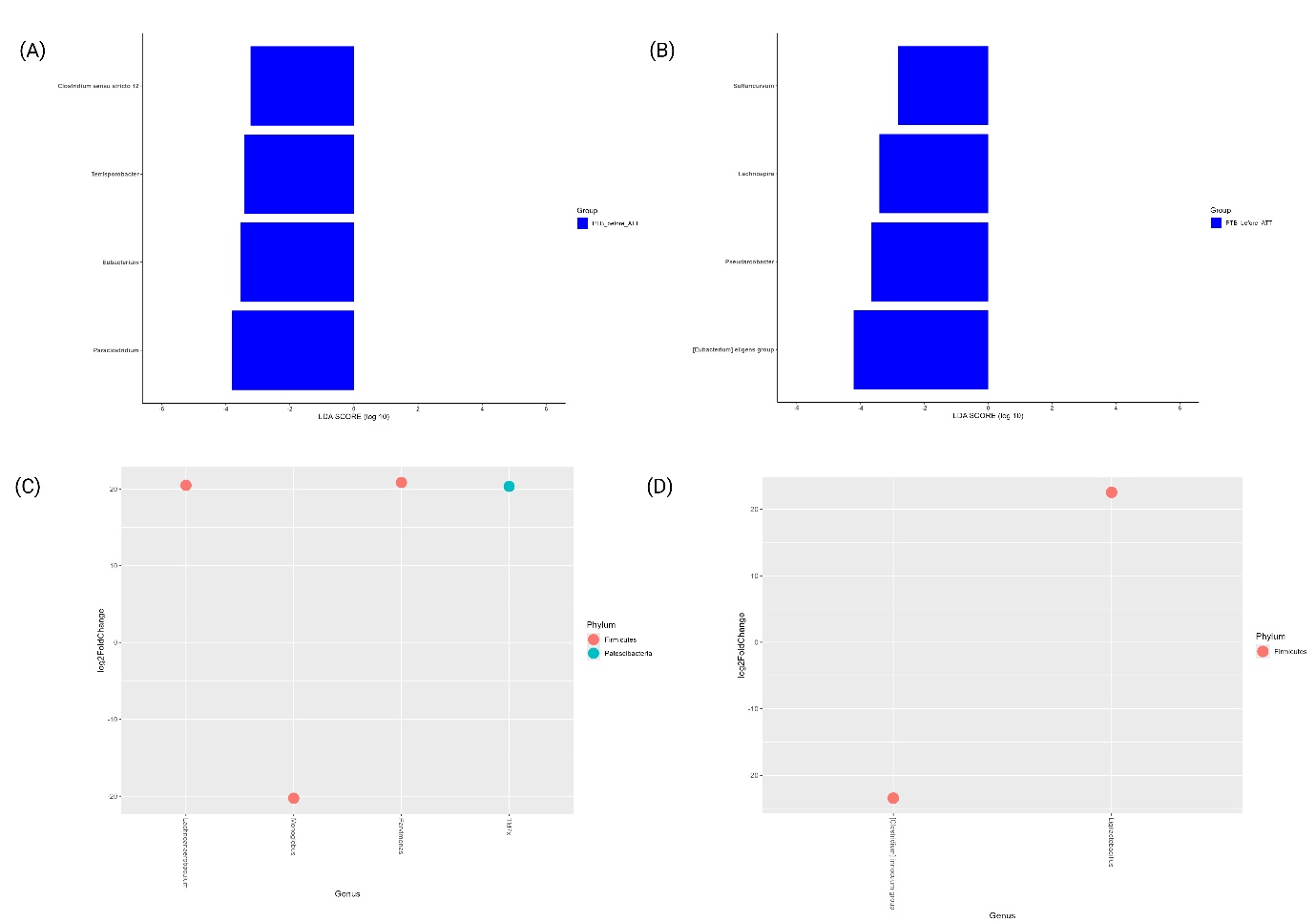
**

**Figure S 5: Differential abundance analysis.** (A) LEfSe analysis for PTB_before_ATT (n=4) Vs. PTB_after_ATT (n=4), (B) LEfSe analysis for PTB_before_ATT (n=5) Vs. PTB_after_ATT_Probiotics (n=5)

**PICRUSt2 analysis for the subgroup analyses for the before and after paired samples**

**
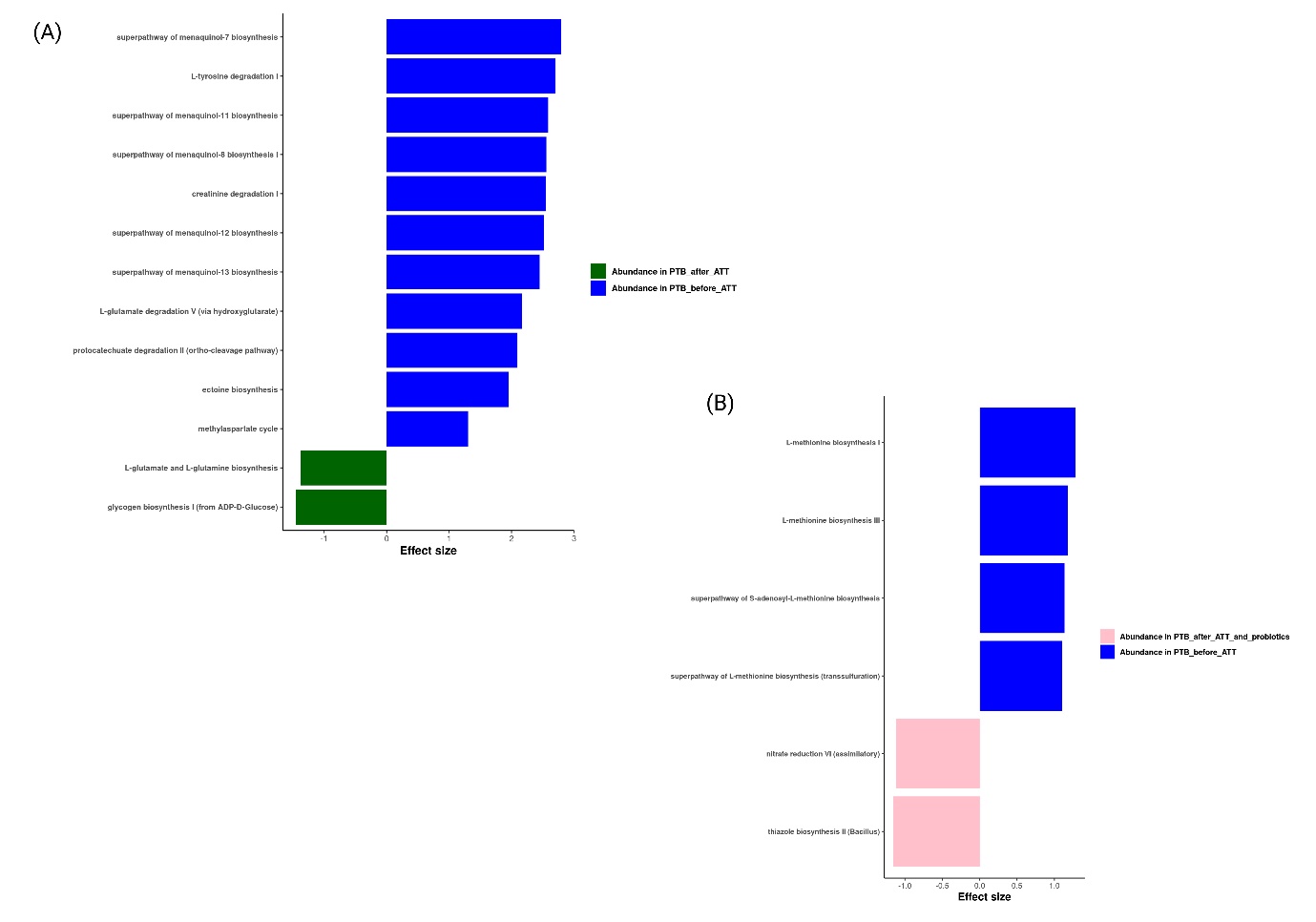
**

**Figure S 6: PICRUSt2 analysis.** (A) PTB_before_ATT (n=4) Vs. PTB_after_ATT (n=4), (B) PTB_before_ATT (n=5) Vs. PTB_after_ATT_Probiotics (n=5)
